# Triglyceride-glucose and TyG-BMI indexes predict acute pancreatitis in adult critically ill patients admitted to intensive care

**DOI:** 10.1016/j.clinsp.2026.101007

**Published:** 2026-06-01

**Authors:** Yong Peng Luo, Meng Ni Lin, Miao Long Zhang, Yong’An Jiang, Luo Feng Pan, Zeng Huang

**Affiliations:** Department of Anesthesiology, Yuhuan People's Hospital, Yuhuan, Zhejiang, China

**Keywords:** Triglyceride-glucose index, Acute pancreatitis, Adult, Restricted cubic splines

## Abstract

**Background:**

The Triglyceride-Glucose Index (TyG) has emerged as a convenient surrogate marker of insulin resistance and has been linked to various metabolic disorders. However, its relationship with Acute Pancreatitis (AP) in adult critically ill patients remains unclear.

**Methods:**

The authors analyzed 2,588 adult critically ill patients from the MIMIC database. Participants were stratified by median TyG and TyG-BMI indices. Logistic regression, restricted cubic spline, and subgroup analyses evaluated associations with AP risk. ROC curves assessed predictive performance.

**Results:**

High TyG and TyG-BMI groups exhibited greater metabolic derangements, longer ICU/hospital stays, and higher comorbidity burden. AP patients (9.4%) had elevated TyG levels (9.9 vs. 8.7, *p* < 0.001). Both indices were independently associated with AP risk in continuous, IQR, and quartile analyses (TyG: OR per IQR = 2.08, 95% CI 1.75–2.48; TyG-BMI: OR per IQR = 1.34, 95% CI 1.12–1.58). TyG showed linear dose-response, whereas TyG-BMI exhibited nonlinear associations. Associations were consistent across subgroups. ROC analysis indicated superior predictive ability for TyG (AUC = 0.809) versus TyG-BMI (AUC = 0.787, *p* = 0.00227).

**Conclusion:**

Both TyG and TyG‑BMI indices are independent predictors of acute pancreatitis, with TyG demonstrating a statistically higher but modestly improved discriminative performance.

## Highlights

• Evidence on TyG and acute pancreatitis risk in critically ill adults is limited.
• This study assessed TyG and TyG-BMI as independent predictors of AP risk.
• Both indices predicted AP; TyG showed linearity and slightly better discrimination.
• TyG may aid early AP risk assessment in ICU, though broader validation is needed.

## Introduction

Acute Pancreatitis (AP) is a common and potentially life-threatening condition in the gastrointestinal tract, characterized by heterogeneous clinical presentations and rapid disease progression.^1^^,^^2^ Severe cases may develop multiorgan dysfunction, leading to significant morbidity and mortality. Epidemiological studies indicate a rising incidence of AP worldwide, with adults bearing a particularly high disease burden.^3^ Traditional risk factors, including gallstones, alcohol consumption, and hypertriglyceridemia, do not fully account for the variation in AP susceptibility, suggesting the involvement of additional metabolic mechanisms.^4^^,^^5^

Metabolic disturbances have increasingly been implicated in pancreatic injury. Hypertriglyceridemia is recognized as a significant cause of AP, accounting for approximately 5%–10% of cases.^6^ Mechanistically, excessive hydrolysis of triglycerides generates free fatty acids, triggering inflammatory responses and pancreatic cytotoxicity.^7^ Insulin resistance, a central feature of metabolic syndrome, has also been shown to exacerbate pancreatic injury through lipid dysregulation, oxidative stress, and inflammatory pathways.^8^^,^^9^ However, a practical and reliable clinical marker of insulin resistance remains a challenge.

The Triglyceride-Glucose Index (TyG) has recently gained attention as a simple, reproducible surrogate for insulin resistance.^10^ Accumulating evidence demonstrates that elevated TyG is associated with diabetes, nonalcoholic fatty liver disease, atherosclerosis, and cardiovascular events.^11^^,^^12^ Emerging studies further suggest that TyG may predict adverse outcomes in hospitalized patients, including infection, renal dysfunction, and multiorgan failure.^13^^,^^14^ Nonetheless, the relationship between TyG and AP has been scarcely investigated. Existing studies are limited to the general population or general hospitalized patients and are not generalizable.^15^ Notably, critically ill patients ‒ a particularly high-risk population ‒ have not been systematically studied.

In this context, the authors hypothesized that elevated TyG and TyG-BMI indices are associated with an increased risk of AP in adult critically ill patients, potentially exhibiting linear, nonlinear, or threshold effects. Leveraging the MIMIC database, the authors applied multivariable logistic regression, restricted cubic splines, piecewise logistic regression, and subgroup analyses to comprehensively assess the relationships between these metabolic indices and AP risk. This study aims to provide robust epidemiological evidence linking metabolic dysregulation to AP in critically ill patients and to evaluate TyG and TyG-BMI as practical tools for early risk stratification.

## Methods

### Study data collection

This study was based on the open-available intensive Monitoring Multi-parameter Intelligent Monitoring IV (MIMIC-IV) database version 3.1, which was a open-free database for researchers. Including the identification of health-related data from patients with the intensive care unit of the Bass Israel Female Deserture Medical Center from 2008 to 2019. These team members completed the training course stipulated by the National Institute of Health (NIH) and the protection of human research participants after the exam, and the authors were allowed to extract data. One author JYA, was approved to use the database. The present research was also approved by the Beth Israel Deaconess Medical Center and the Massachusetts Institute of Technology (Cambridge, Massachusetts, in the United States). The information of all the subjects was anonymous and did not require the consent of the participants. certificate number was: 58572169. Structural Query Language (SQL) was used to extract all patient information from the MIMIC-IV database. The design, conduct, and reporting of this retrospective cohort study adhered to the STROBE Statement guidelines for observational studies.

### Screening of patient study populations

**Fig. 1:**
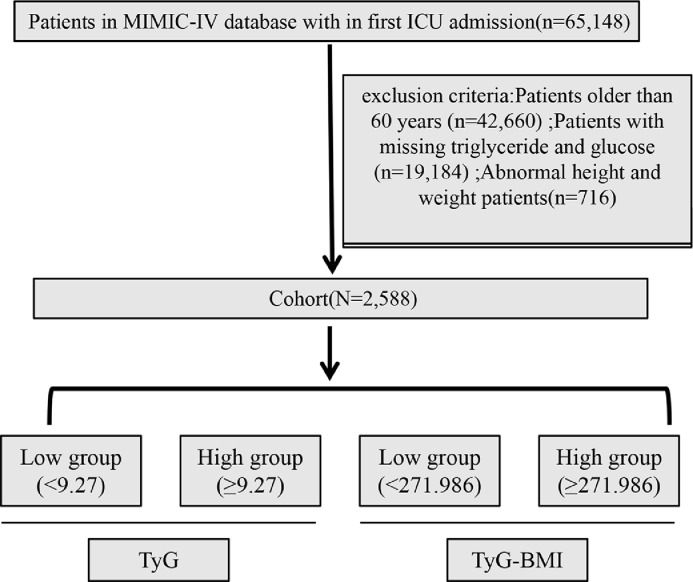
Flowchart of this study.

Intensive care patients were identified according to ICD‑9 and ICD‑10 disease codes (K85 and 5770). Because the primary diagnosis may not always be listed first, the authors included records if the disease appeared within the first five diagnoses. The exclusion criteria were as follows: 1) Patients younger than 18-years or older than 59-years at the time of first ICU admission; 2) Patients with multiple ICU admissions, for whom only the first admission was analyzed; 3) Patients with an ICU stay shorter than 3-hours; and 4) Patients lacking Triglyceride (TG) or Fasting Blood Glucose (FBG) data on the first day of admission. Fig. 1 presents the complete flow chart of patient selection. After screening 65,148 critically ill patients admitted to the ICU, a total of 8,858 adult patients were included according to the above criteria.

### Variable data extraction using structured query language (SQL)

SQL was used to extract all data from the MIMIC-IV database, with only the first variable being used for analysis. The extraction code is available from: https://github.com/MIT-LCP/mimic-website. The variables used were: 1) Demographic data: age, sex, height (cm), weight (kg), marital status, and race; 2) Laboratory test results: Glucose (Glu, mg/dL), Triglycerides (TG, mg/dL), Blood urea nitrogen (Bun, mg/dL), serum potassium (K, mEq/L), Creatinine (Cre, mg/dL), total bilirubin (mg/dL), Lactate Dehydrogenase (LDH, IU/L), neutrophil % (%), Red Blood Cells (RBC, m/μL), White Blood Cells (WBC, K/μL), Platelets (PLT, K/μL), and Hemoglobin (Hb, g/dL). 3) Patient vital signs: Heart Rate (HR, bpm), Systolic Blood Pressure (SBP, mmHg), Diastolic Blood Pressure (DBP, mmHg), Respiratory Rate (RR, bpm), and body temperature (°C). 4) Complications: atrial fibrillation, acute myocardial infarction, congestive heart failure, hypertension, malignancy, ascites, severe liver disease, chronic obstructive pulmonary disease, rheumatic disease, hypothyroidism, hyperlipidemia, chronic kidney disease, and acute pancreatitis. 5) Disease severity: Logistic Organ Dysfunction Score (LODS), Abbreviated Acute Physiology Score II (APSIII), and Systemic Inflammatory Response Syndrome (SIRS). Follow-up was from admission to death. The primary outcome was the incidence of acute pancreatitis, and secondary outcomes were length of hospital stay and ICU stay.

### Calculation of triglyceride glucose (TyG) and the triglyceride glucose body mass index

The TyG index is a key indicator and is calculated as: ln [triglyceride (mg/dL) × glucose (mg/dL)/2]. This index is a fundamental parameter in the assessment process.

The TyG-BMI index is calculated as TyG index × BMI index. TyG and TyG‑BMI indices were calculated from triglyceride and glucose values obtained at ICU admission, before AP diagnosis.

### Managing missing data for patients in this study

Missing data in the MIMIC dataset were handled rigorously. Variables with a missing value prevalence exceeding 50% were excluded from the study to ensure the integrity of the analysis. For variables with a missing value prevalence below 20%, a robust method combining multiple interpolation techniques was used to meticulously impute missing values while maintaining data consistency and reliability (All variables that need to be interpolated are listed in Table S1).

### Statistical analysis

This study included adult critically ill patients, who were stratified by the median TyG index into Low (< 9.27) and High (≥ 9.27) groups. Similarly, patients were categorized by the TyG-BMI index as Low (< 271.986) and High (≥ 271.986). Continuous variables were expressed as mean ± Standard Deviation (SD) or median (Interquartile Range, IQR), whereas categorical variables were presented as counts and percentages. Differences between groups were assessed using Fisher’s exact test or Pearson’s Chi-Square test for categorical variables, and the Mann-Whitney *U* test or Kruskal-Wallis test for continuous variables, as appropriate.

The association between the TyG index and the risk of acute pancreatitis was evaluated using multivariable logistic regression models, and results were reported as Odds Ratios (ORs) with corresponding 95% Confidence Intervals (95% CIs). To minimize multicollinearity, covariates were progressively adjusted across four models. Model 0 included no covariate adjustment; Model I adjusted for demographic characteristics (age, sex, marital status, and race; height and weight were additionally included in TyG analyses but not in TyG-BMI models to avoid collinearity); Model II further adjusted for vital signs (heart rate, systolic and diastolic blood pressure, respiratory rate, and temperature); Model III incorporated biochemical parameters (potassium, creatinine, total bilirubin, lactate dehydrogenase, neutrophil count, white blood cell count, red blood cell count, platelet count, and hemoglobin); and Model IV included Model III variables plus clinical comorbidities and severity indices (APSIII, LODS, SIRS, atrial fibrillation, acute myocardial infarction, congestive heart failure, hypertension, malignant neoplasm, ascites, severe liver disease, chronic obstructive pulmonary disease, rheumatic disease, hypothyroidism, hyperlipidemia, and chronic kidney disease). Both continuous and quartile-based TyG indices were analyzed, and p-values for trend were computed across quartiles.

To evaluate potential nonlinear relationships, generalized additive models were fitted after adjusting for the same covariates. Threshold effect analyses were conducted using segmented logistic regression models, log-likelihood ratio tests, and bootstrap resampling to identify specific TyG thresholds associated with acute pancreatitis risk. Odds ratios and 95% CIs were estimated separately for each segment. Restricted Cubic Spline (RCS) logistic regression was also employed to flexibly model nonlinear associations between continuous TyG levels and dichotomous outcomes. Three knots were placed at the 25^th^, 50^th^, and 75^th^ percentiles of the exposure distribution, and Wald chi-square tests were used to evaluate both overall and nonlinear associations. The first derivative of the predicted values from the RCS model was examined to identify potential inflection points, restricting the analysis to the 5^th^–95^th^ percentile range of exposure. When a turning point was detected, segmented logistic regression was used to estimate aORs and 95% CIs on either side of the threshold for comparison with overall model estimates.

Subgroup and interaction analyses were performed to assess the robustness of associations across population strata. Categorical variables (including sex, hypertension, diabetes, and smoking status) were used as subgrouping factors. Within each subgroup, adjusted logistic regression models were independently fitted to estimate aORs and 95% CIs for the association between the TyG index (or TyG-BMI) and acute pancreatitis. Interaction terms (TyG × subgroup or TyG-BMI × subgroup) were then introduced into the overall regression model to test for statistical interaction, with p-values for interaction reported accordingly. Forest plots were generated to visualize the associations and interactions across subgroups (sex, race, marital status, atrial fibrillation, hypertension, malignant neoplasm, hyperlipidemia, hypothyroidism, and chronic kidney disease), displaying the adjusted aORs and 95% CIs along with sample sizes.

All statistical analyses were conducted using R software (version 4.2.3; R Foundation for Statistical Computing, Vienna, Austria). All reported p-values were two-sided, and a value of *p* < 0.05 was considered statistically significant.

## Results

### Baseline characteristics of critically ill patients grouped by median Tyg and TyG-BMI index

This study enrolled 2,588 adult patients in critical care from the MIMIC database (The inclusion and exclusion criteria process is shown in Fig. 1.). They were first stratified by their median TyG index (9.27) (Table 1). Compared with participants in the low TyG group, those with a high TyG index exhibited higher body weight (median 92.0 vs. 81.3 kg, *p* < 0.001) and BMI (31.0 vs. 27.8 kg/m^2^, *p* < 0.001), as well as a greater proportion of males (67.0% vs. 61.0%, *p* = 0.002). Clinically, the high TyG group presented with significantly longer hospital stays (16.0 vs. 10.8 days, *p* < 0.001) and ICU stays (8.2 vs. 4.8 days, *p* < 0.001). They also had a higher heart rate (98 vs. 90 bpm, *p* < 0.001) and respiratory rate (21 vs. 20 bpm, *p* < 0.001). Laboratory parameters revealed that the high TyG group had markedly elevated fasting glucose (154 vs. 112 mg/dL), triglycerides (259.5 vs. 103.0 mg/dL), blood urea nitrogen (18.0 vs. 14.0 mg/dL), creatinine (1.0 vs. 0.9 mg/dL), and LDH (389.5 vs. 323.0 IU/L), all with *p* < 0.001.

**Table 1:** Baseline characteristics of adult’s patients according to median TyG index.

	Level	Overall	High (TyG index ≥ 9.27)	Low (TyG index < 9.27)	p-value
n		2,588	1,294	1,294	
Admission age (median [IQR], years)		49.0 [39.2, 54.9]	48.4 [38.9, 54.7]	50.0 [39.5, 55.2]	0.089
Gender (%)	Female	932 (36.0)	427 (33.0)	505 (39.0)	0.002
	Male	1656 (64.0)	867 (67.0)	789 (61.0)	
Height (median [IQR], cm)		173.0 [165.0, 178.0]	173.0 [165.0, 180.0]	170.2 [163.0, 178.0]	0.007
Weight (median [IQR], kg)		87.0 [71.8, 104.5]	92.0 [76.2, 110.0]	81.3 [68.0, 99.1]	<0.001
Race (%)	Blacks	316 (12.2)	158 (12.2)	158 (12.2)	0.052
	Others	931 (36.0)	494 (38.2)	437 (33.8)	
	Whites	1,341 (51.8)	642 (49.6)	699 (54.0)	
Marital status (%)	Divorced	161 (6.2)	70 (5.4)	91 (7.0)	0.046
	Married	871 (33.7)	418 (32.3)	453 (35.0)	
	Others	1,556 (60.1)	806 (62.3)	750 (58.0)	
Los hospital (median [IQR], days)		13.4 [5.9, 25.0]	16.0 [7.4, 28.7]	10.8 [5.0, 21.3]	<0.001
Los ICU (median [IQR], days)		6.1 [2.4, 13.3]	8.2 [3.1, 16.1]	4.8 [2.0, 10.7]	<0.001
Heart rate (median [IQR], bpm)		94.0 [80.0, 110.0]	98.0 [83.0, 113.0]	90.0 [76.0, 105.0]	<0.001
SBP (median [IQR], mmHg)		124.0 [109.0, 140.0]	124.0 [108.0, 140.0]	124.0 [109.0, 140.0]	0.763
DBP (median [IQR], mmHg)		74.0 [63.0, 86.0]	73.0 [63.0, 86.0]	75.0 [63.0, 86.0]	0.288
Respiratory rate (median [IQR], bpm)		20.0 [17.0, 25.0]	21.0 [17.0, 26.0]	20.0 [16.0, 23.9]	<0.001
Temperature (median [IQR],°C)		36.9 [36.6, 37.3]	36.9 [36.6, 37.4]	36.9 [36.6, 37.2]	0.001
Bun (median [IQR], mg/dL)		16.0 [11.0, 25.0]	18.0 [12.0, 30.0]	14.0 [10.0, 21.0]	<0.001
Potassium (median [IQR], mEq/L)		4.1 [3.7, 4.5]	4.2 [3.8, 4.7]	4.0 [3.7, 4.4]	<0.001
Creatinine (median [IQR], mg/dL)		0.9 [0.7, 1.4]	1.0 [0.8, 1.8]	0.9 [0.7, 1.2]	<0.001
Total bilirubin (median [IQR], mg/dL)		0.7 [0.4, 1.5]	0.7 [0.4, 1.5]	0.7 [0.4, 1.4]	0.420
LDH (median [IQR], IU/L)		351.0 [234.0, 591.0]	389.5 [263.0, 658.0]	323.0 [218.0, 522.8]	<0.001
Neutrophils (median [IQR], %)		81.0 [73.2, 87.1]	81.2 [74.0, 87.8]	81.0 [72.8, 87.0]	0.056
WBC (median [IQR], %)		11.4 [8.0, 16.2]	12.2 [8.3, 17.3]	10.8 [7.7, 15.1]	<0.001
RBC (median [IQR], %)		4.0 [3.3, 4.5]	4.0 [3.3, 4.5]	4.0 [3.2, 4.5]	0.909
Platelet (median [IQR], 10^9^/L)		203.5 [141.0, 267.0]	200.0 [138.2, 266.0]	206.0 [145.0, 268.8]	0.374
Hemoglobin (median [IQR], g/dL)		11.8 [9.8, 13.5]	11.8 [9.8, 13.5]	11.9 [9.7, 13.5]	0.977
APSIII (median [IQR])		44.0 [30.0, 64.0]	49.0 [34.2, 71.0]	38.0 [27.0, 57.0]	<0.001
LODS (median [IQR])		5.0 [2.0, 8.0]	6.0 [4.0, 8.8]	4.0 [2.0, 7.0]	<0.001
SIRS (median [IQR])		3.0 [2.0, 4.0]	3.0 [2.0, 4.0]	3.0 [2.0, 3.0]	<0.001
Glucose (median [IQR], mg/dL)		129.0 [104.0, 171.0]	154.0 [122.0, 216.0]	112.0 [96.0, 135.0]	<0.001
Triglycerides (median [IQR], mg/dL)		151.5 [99.0, 263.0]	259.5 [180.0, 401.5]	103.0 [76.0, 135.0]	<0.001
Atrial Fibrillation (%)	No	2,306 (89.1)	1,158 (89.5)	1,148 (88.7)	0.570
	Yes	282 (10.9)	136 (10.5)	146 (11.3)	
Acute Myocardial Infarction (%)	No	2,523 (97.5)	1,261 (97.4)	1,262 (97.5)	1.000
	Yes	65 (2.5)	33 (2.6)	32 (2.5)	
Congestive Heart Failure (%)	No	2,215 (85.6)	1,107 (85.5)	1,108 (85.6)	1.000
	Yes	373 (14.4)	187 (14.5)	186 (14.4)	
Hypertension (%)	No	1,432 (55.3)	673 (52.0)	759 (58.7)	0.001
	Yes	1,156 (44.7)	621 (48.0)	535 (41.3)	
Malignant Neoplasm (%)	No	2,371 (91.6)	1,201 (92.8)	1,170 (90.4)	0.033
	Yes	217 (8.4)	93 (7.2)	124 (9.6)	
Ascites (%)	No	2,215 (85.6)	1,133 (87.6)	1,082 (83.6)	0.005
	Yes	373 (14.4)	161 (12.4)	212 (16.4)	
Severe Liver Disease (%)	No	1,969 (76.1)	998 (77.1)	971 (75.0)	0.231
	Yes	619 (23.9)	296 (22.9)	323 (25.0)	
COPD (%)	No	2,462 (95.1)	1,223 (94.5)	1,239 (95.7)	0.171
	Yes	126 (4.9)	71 (5.5)	55 (4.3)	
Rheumatic Disease (%)	No	2,524 (97.5)	1,258 (97.2)	1,266 (97.8)	0.376
	Yes	64 (2.5)	36 (2.8)	28 (2.2)	
Hypothyroidism (%)	No	2413 (93.2)	1219 (94.2)	1194 (92.3)	0.060
	Yes	175 (6.8)	75 (5.8)	100 (7.7)	
Hyperlipidemia (%)	No	2041 (78.9)	1003 (77.5)	1038 (80.2)	0.102
	Yes	547 (21.1)	291 (22.5)	256 (19.8)	
Chronic Kidney Disease (%)	No	2345 (90.6)	1159 (89.6)	1186 (91.7)	0.080
	Yes	243 (9.4)	135 (10.4)	108 (8.3)	
Acute Pancreatitis (%)	No	2,346 (90.6)	1,128 (87.2)	1,218 (94.1)	<0.001
	Yes	242 (9.4)	166 (12.8)	76 (5.9)	
TyG (median [IQR])		9.3 [8.7, 9.9]	9.9 [9.5, 10.4]	8.7 [8.4, 9.0]	<0.001
BMI (median [IQR], kg/m^2^)		29.3 [25.0, 34.7]	31.0 [26.2, 36.7]	27.8 [23.8, 32.8]	<0.001

TyG, Triglyceride-Glucose; IQR, Interquartile Range; BMI, Body Mass Index; LDH, Lactate Dehydrogenase; RBC, Red Blood Cell; WBC, White Blood Cell; COPD, Chronic Obstructive Pulmonary Disease; APSIII, Acute Physiology Score III; LODS, Logistic Organ Dysfunction System; SIRS, Systemic Inflammatory Response Syndrome.

In terms of comorbidities, hypertension (48.0% vs. 41.3%, *p* = 0.001) and acute pancreatitis (12.8% vs. 5.9%, *p* < 0.001) were more frequent among individuals with high TyG levels. No significant differences were observed in the prevalence of atrial fibrillation, myocardial infarction, or congestive heart failure. Moreover, disease severity scores such as APSIII (49 vs. 38) and LODS (6 vs. 4) were significantly higher in the high TyG group (*p* < 0.001 for both), suggesting a greater systemic burden and disease acuity.

When participants were stratified by the median TyG-BMI index (271.986) (Table S2), similar trends were observed. Individuals in the high TyG-BMI group had markedly higher body weight (104.0 vs. 73.0 kg, *p* < 0.001) and BMI (34.7 vs. 25.0 kg/m^2^, *p* < 0.001). They also exhibited elevated heart rate (96 vs. 92 bpm, *p* < 0.001), respiratory rate (21 vs. 19 bpm, *p* < 0.001), BUN (17.0 vs. 14.0 mg/dL, *p* < 0.001), and creatinine (1.0 vs. 0.9 mg/dL, *p* < 0.001). Consistent with metabolic overload, both glucose (141 vs. 118 mg/dL) and triglyceride (213 vs. 120 mg/dL) levels were substantially higher in the high TyG-BMI group (*p* < 0.001). Participants in this group also showed longer hospitalization (14.2 vs. 12.7 days, *p* = 0.005), higher APSIII (48 vs. 40, *p* < 0.001), and LODS (5 vs. 4, *p* < 0.001).

Regarding comorbidities, high TyG-BMI was significantly associated with increased prevalence of hypertension (52.4% vs. 36.9%, *p* < 0.001), congestive heart failure (16.5% vs. 12.4%, *p* = 0.004), COPD (6.3% vs. 3.5%, *p* = 0.001), and acute pancreatitis (12.1% vs. 6.6%, *p* < 0.001). Conversely, hyperlipidemia was more common in the high TyG-BMI group (24.2% vs. 18.1%, *p* < 0.001).

### Baseline characteristics of patients with and without acute pancreatitis

Among 2,588 patients (Table 2), 242 (9.4%) were diagnosed with AP. Compared with those without AP, patients with AP were younger (*p* = 0.002) and had longer hospital stays (17.7 vs. 13.0 days, *p* < 0.001). They also showed higher heart and respiratory rates and slightly elevated temperature (all *p* < 0.01).

**Table 2:** Baseline characteristics of acute pancreatitis.

Characteristics	Level	Overall	No	Yes	p
n		2,588	2,346	242	
Admission age (median [IQR], years)		49.0 [39.2, 54.9]	49.4 [39.5, 55.1]	46.2 [36.6, 53.2]	0.002
Gender (%)	F	932 (36.0)	846 (36.1)	86 (35.5)	0.927
	M	1,656 (64.0)	1,500 (63.9)	156 (64.5)	
Height (median [IQR], cm)		173.0 [165.0, 178.0]	173.0 [164.2, 178.0]	173.0 [165.0, 179.5]	0.819
Weight (median [IQR], kg)		87.0 [71.8, 104.5]	86.7 [71.5, 104.4]	88.7 [75.8, 105.8]	0.105
Race (%)	Blacks	316 (12.2)	291 (12.4)	25 (10.3)	0.140
	Others	931 (36.0)	854 (36.4)	77 (31.8)	
	Whites	1,341 (51.8)	1,201 (51.2)	140 (57.9)	
Marital status (%)	Divorced	161 (6.2)	144 (6.1)	17 (7.0)	0.284
	Married	871 (33.7)	780 (33.2)	91 (37.6)	
	Others	1,556 (60.1)	1,422 (60.6)	134 (55.4)	
Los hospital (median [IQR], days)		13.4 [5.9, 25.0]	13.0 [5.8, 24.6]	17.7 [9.6, 27.5]	<0.001
Los ICU (median [IQR], days)		6.1 [2.4, 13.3]	6.1 [2.4, 13.0]	6.7 [2.8, 15.9]	0.068
Heart rate (median [IQR], bpm)		94.0 [80.0, 110.0]	93.0 [79.0, 108.0]	109.0 [92.2, 123.0]	<0.001
SBP (median [IQR], mmHg)		124.0 [109.0, 140.0]	124.0 [109.0, 140.0]	124.0 [110.0, 143.0]	0.359
DBP (median [IQR], mmHg)		74.0 [63.0, 86.0]	74.0 [63.0, 86.0]	75.0 [62.2, 88.0]	0.355
Respiratory rate (median [IQR], bpm)		20.0 [17.0, 25.0]	20.0 [16.0, 24.0]	22.0 [18.0, 27.0]	<0.001
Temperature (median [IQR],°C)		36.9 [36.6, 37.3]	36.9 [36.6, 37.3]	37.0 [36.6, 37.6]	0.001
Bun (median [IQR], mg/dL)		16.0 [11.0, 25.0]	16.0 [11.0, 25.0]	15.0 [10.0, 27.0]	0.352
Potassium (median [IQR], mEq/L)		4.1 [3.7, 4.5]	4.1 [3.7, 4.5]	4.1 [3.7, 4.6]	0.705
Creatinine (median [IQR], mg/dL)		0.9 [0.7, 1.4]	0.9 [0.7, 1.4]	1.0 [0.7, 1.9]	0.701
Total bilirubin (median [IQR], mg/dL)		0.7 [0.4, 1.5]	0.6 [0.4, 1.4]	0.9 [0.5, 2.4]	<0.001
LDH (median [IQR], IU/L)		351.0 [234.0, 591.0]	346.0 [230.0, 588.5]	392.5 [283.0, 638.0]	0.011
Neutrophils (median [IQR], %)		81.0 [73.2, 87.1]	81.0 [73.0, 87.0]	82.7 [75.0, 88.4]	0.014
WBC (median [IQR], %)		11.4 [8.0, 16.2]	11.3 [7.9, 15.9]	12.8 [8.8, 18.5]	0.003
RBC (median [IQR], %)		4.0 [3.3, 4.5]	4.0 [3.3, 4.5]	3.7 [3.1, 4.3]	0.002
Platelet (median [IQR], 10^9^/L)		203.5 [141.0, 267.0]	205.0 [143.0, 267.0]	185.0 [128.5, 266.0]	0.280
Hemoglobin (median [IQR], g/dL)		11.8 [9.8, 13.5]	11.8 [9.8, 13.5]	11.2 [9.6, 13.6]	0.464
APSIII (median [IQR])		44.0 [30.0, 64.0]	43.0 [29.0, 63.0]	51.0 [36.0, 75.0]	<0.001
LODS (median [IQR])		5.0 [2.0, 8.0]	5.0 [3.0, 8.0]	5.0 [2.0, 8.0]	0.643
SIRS (median [IQR])		3.0 [2.0, 4.0]	3.0 [2.0, 3.0]	3.0 [3.0, 4.0]	<0.001
Glucose (median [IQR], mg/dL)		129.0 [104.0, 171.0]	128.0 [104.0, 170.0]	133.5 [104.0, 178.5]	0.418
Triglycerides (median [IQR], mg/dL)		151.5 [99.0, 263.0]	147.0 [96.0, 248.0]	243.0 [147.2, 521.0]	<0.001
Atrial Fibrillation (%)	No	2306 (89.1)	2087 (89.0)	219 (90.5)	0.534
	Yes	282 (10.9)	259 (11.0)	23 (9.5)	
Acute Myocardial Infarction (%)	No	2523 (97.5)	2281 (97.2)	242 (100.0)	0.016
	Yes	65 (2.5)	65 (2.8)	0 (0.0)	
Congestive Heart Failure (%)	No	2215 (85.6)	1988 (84.7)	227 (93.8)	<0.001
	Yes	373 (14.4)	358 (15.3)	15 (6.2)	
Hypertension (%)	No	1432 (55.3)	1305 (55.6)	127 (52.5)	0.384
	Yes	1156 (44.7)	1041 (44.4)	115 (47.5)	
Malignant Neoplasm (%)	No	2371 (91.6)	2148 (91.6)	223 (92.1)	0.847
	Yes	217 (8.4)	198 (8.4)	19 (7.9)	
Ascites (%)	No	2215 (85.6)	2045 (87.2)	170 (70.2)	<0.001
	Yes	373 (14.4)	301 (12.8)	72 (29.8)	
Severe Liver Disease (%)	No	1969 (76.1)	1817 (77.5)	152 (62.8)	<0.001
	Yes	619 (23.9)	529 (22.5)	90 (37.2)	
COPD (%)	No	2462 (95.1)	2229 (95.0)	233 (96.3)	0.474
	Yes	126 (4.9)	117 (5.0)	9 (3.7)	
Rheumatic Disease (%)	No	2524 (97.5)	2288 (97.5)	236 (97.5)	1.000
	Yes	64 (2.5)	58 (2.5)	6 (2.5)	
Hypothyroidism (%)	No	2413 (93.2)	2186 (93.2)	227 (93.8)	0.816
	Yes	175 (6.8)	160 (6.8)	15 (6.2)	
Hyperlipidemia (%)	No	2041 (78.9)	1841 (78.5)	200 (82.6)	0.153
	Yes	547 (21.1)	505 (21.5)	42 (17.4)	
Chronic Kidney Disease (%)	No	2345 (90.6)	2118 (90.3)	227 (93.8)	0.095
	Yes	243 (9.4)	228 (9.7)	15 (6.2)	
TyG (median [IQR])		9.3 [8.7, 9.9]	9.2 [8.7, 9.8]	9.8 [9.1, 10.7]	<0.001

TyG, Triglyceride-Glucose; IQR, Interquartile Range; BMI, Body Mass Index; LDH, Lactate Dehydrogenase; RBC, Red Blood Cell; WBC, White Blood Cell; COPD, Chronic Obstructive Pulmonary Disease; APSIII, Acute Physiology Score III; LODS, Logistic Organ Dysfunction System; SIRS, Systemic Inflammatory Response Syndrome.

Laboratory findings indicated greater metabolic and inflammatory disturbances in the AP group, including higher levels of total bilirubin, LDH, WBC count, and triglycerides (all *p* < 0.01), along with lower red blood cell counts (*p* = 0.002). The APSIII and SIRS scores were significantly increased (both *p* < 0.001), whereas LODS scores showed no difference.

Notably, the TyG index was markedly elevated in AP patients (9.8 vs. 9.2, *p* < 0.001), suggesting a strong link between insulin resistance and AP onset. In terms of comorbidities, AP patients more frequently had ascites and severe liver disease but were less likely to have congestive heart failure or myocardial infarction (all *p* < 0.05).

### Association between the TyG and TyG-BMI indices and the risk of acute pancreatitis

Multivariable logistic regression analyses revealed that both the TyG and TyG-BMI indices were significantly and positively associated with the occurrence of acute pancreatitis in all models (*p* < 0.001) (Table 3). When analyzed as continuous variables, each one-unit increase in the TyG index was associated with a markedly higher risk of AP (Model 0: OR = 1.95, 95% CI 1.72–2.21; Model 4: adjusted OR(aOR) = 1.87, 95% CI 1.61–2.17, *p* < 0.001). When standardized per Interquartile Range (IQR), each IQR increment in TyG corresponded to a 2.08-fold higher risk of AP (95% CI 1.75–2.48, *p* < 0.001), indicating a stable and dose-dependent relationship.

**Table 3:** Logistic regression analyses for the correlation between the TyG/TyG-BMI index and the occurrence of acute pancreatitis among adults populations.

Exposure	Model 0	Model 1	Model 2	Model 3	Model 4
	aOR (95% CI); p	aOR (95% CI); p	aOR (95% CI); p	aOR (95% CI); p	aOR (95% CI); p
Continuous variable	1.95 (1.72‒2.21); *p* < 0.001	1.98 (1.74‒2.25); *p* < 0.001	1.80 (1.58‒2.06); *p* < 0.001	1.88 (1.63‒2.16); *p* < 0.001	1.87 (1.61‒2.17); *p* < 0.001
per IQR increase in TyG	2.18 (1.88‒2.53); *p* < 0.001	2.22 (1.91‒2.59); *p* < 0.001	1.99 (1.71‒2.33); *p* < 0.001	2.09 (1.77‒2.46); *p* < 0.001	2.08 (1.75‒2.48); *p* < 0.001
TyG quartile					
Q1	Ref	Ref	Ref	Ref	Ref
Q2	1.67 (1.04‒2.71); *p* = 0.035	1.73 (1.08‒2.83); *p* = 0.024	1.60 (0.99‒2.64); *p* = 0.057	1.67 (1.02‒2.77); *p* = 0.042	1.68 (1.02‒2.80); *p* = 0.045
Q3	1.86 (1.18‒3.01); *p* = 0.009	1.97 (1.24‒3.19); *p* = 0.005	1.66 (1.03‒2.71); *p* = 0.039	1.72 (1.06‒2.84); *p* = 0.029	1.78 (1.09‒2.97); *p* = 0.024
Q4	4.56 (3.03‒7.08); *p* < 0.001	4.95 (3.25‒7.76); *p* < 0.001	3.99 (2.59‒6.31); *p* < 0.001	4.56 (2.92‒7.31); *p* < 0.001	4.67 (2.93‒7.63); *p* < 0.001
p for trend	1.65 (1.46‒1.89); *p* < 0.001	1.69 (1.49‒1.94); *p* < 0.001	1.58 (1.38‒1.81); *p* < 0.001	1.64 (1.43‒1.90); *p* < 0.001	1.65 (1.43‒1.92); *p* < 0.001
TyG-BMI	1.0020 (1.00‒1.00); *p* < 0.001	1.0021 (1.00‒1.00); *p* < 0.001	1.0015 (1.00‒1.00); *p* = 0.022	1.0023 (1.00‒1.00); *p* < 0.001	1.0025 (1.00‒1.00); *p* < 0.001
Per IQR increase in TyG-BMI	1.2530 (1.09‒1.43); *p* < 0.001	1.2646 (1.10‒1.44); *p* < 0.001	1.1814 (1.02‒1.36); *p* = 0.022	1.3020 (1.11‒1.52); *p* < 0.001	1.3351 (1.12‒1.58); *p* < 0.001
TyG-BMI quartile					
Q1	Ref	Ref	Ref	Ref	Ref
Q2	1.5757 (1.01‒2.48); *p* = 0.046	1.6286 (1.04‒2.57); *p* = 0.034	1.4350 (0.91‒2.28); *p* = 0.122	1.5225 (0.96‒2.44); *p* = 0.077	1.4570 (0.91‒2.36); *p* = 0.121
Q3	2.3640 (1.56‒3.64); *p* < 0.001	2.4910 (1.64‒3.85); *p* < 0.001	2.1327 (1.39‒3.32); *p* < 0.001	2.2991 (1.49‒3.62); *p* < 0.001	2.4220 (1.55‒3.86); *p* < 0.001
Q4	2.5802 (1.72‒3.96); *p* < 0.001	2.7568 (1.83‒4.24); *p* < 0.001	2.2071 (1.45‒3.43); *p* < 0.001	2.6563 (1.71‒4.21); *p* < 0.001	2.5865 (1.63‒4.18); *p* < 0.001
p for trend	1.3578 (1.20‒1.54); *p* < 0.001	1.3860 (1.23‒1.57); *p* < 0.001	1.2976 (1.14‒1.48); *p* < 0.001	1.3802 (1.21‒1.58); *p* < 0.001	1.3820 (1.20‒1.60); *p* < 0.001

TyG, Triglyceride-Glucose; IQR, Interquartile Range; BMI, Body Mass Index; aOR, adjusted Odds Ratio; 95% CI, 95% Confidence Interval; TyG index: Q1 (5.94–8.73), Q2 (8.73–9.27), Q3 (9.27–9.89), Q4 (9.90–13.9); TyG-BMI index: Q1 (94.2–225), Q2 (225–272), Q3 (272–339), Q4 (340–1022). Model 0: Unadjusted. Model 1: Admission age, gender, race, marital status (The BMI index will be additionally adjusted for the TyG index).

Model 2: In addition to the variables in Model 1, add heart rate, SBP, DBP, respiratory rate, temperature. Model 3: In addition to the variables in Model 2, add potassium, creatinine, total bilirubin, LDH, neutrophils, WBC, RBC, platelet, hemoglobin. Model 4: In addition to the variables in Model 3, add APSIII, LODS, SIRS, atrial fibrillation, acute myocardial infarction, congestive heart failure, hypertension, malignant neoplasm, ascites, severe liver disease, COPD, rheumatic disease, hypothyroidism, hyperlipidemia, chronic kidney disease.

When stratified by quartiles, the incidence of AP increased progressively across TyG quartiles (p for trend < 0.001). Compared with participants in the lowest Quartile (Q1), the risk of AP rose sequentially in Q2, Q3, and Q4 (Q2: aOR = 1.68, 95% CI 1.02–2.80; Q3: aOR = 1.78, 95% CI 1.09–2.97; Q4: aOR = 4.67, 95% CI 2.93–7.63; all *p* < 0.001). This trend remained statistically significant even in the fully adjusted model, suggesting that a higher TyG index is an independent risk factor for acute pancreatitis. Similarly, the TyG-BMI index exhibited a consistent positive association with AP risk. Each unit increase in TyG-BMI was independently associated with a higher likelihood of AP (aOR = 1.0025, 95% CI 1.00–1.00, *p* < 0.001), and each IQR increment corresponded to a 1.34-fold increase in risk (95% CI 1.12–1.58, *p* < 0.001). When categorized into quartiles, participants in Q3 and Q4 demonstrated significantly elevated risks compared with those in Q1 (Q3: aOR = 2.42, 95% CI 1.55–3.86; Q4: aOR = 2.59, 95% CI 1.63–4.18; both *p* < 0.001), with a clear linear trend (p for trend < 0.001). Notably, whether assessed by TyG or TyG-BMI, both indices showed a stable and linear increase in AP risk. The effect estimates remained nearly unchanged across progressively adjusted models (Models 0–4), underscoring the robustness and independence of these associations.

### Dose-response and nonlinear associations of TyG and TyG-BMI indices with acute pancreatitis risk

**Fig. 2:**
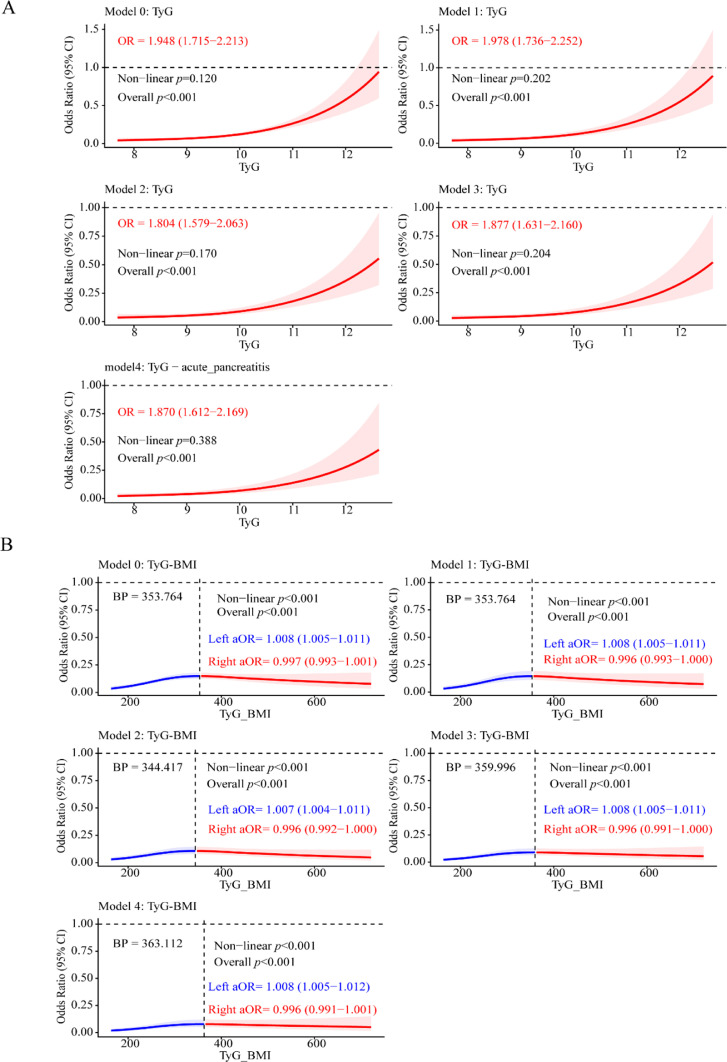
Restricted cubic spline analysis of the association between TyG index, TyG-BMI index, and the risk of acute pancreatitis. (A) The Restricted Cubic Spline (RCS) model illustrates a linear relationship between the TyG index and the risk of Acute Pancreatitis (AP). (B) A nonlinear association was observed between the TyG-BMI index and AP risk. The solid line represents the adjusted Odds Ratio (OR), and the shaded area denotes the 95% Confidence Interval (95% CI). The reference value (OR = 1.0) corresponds to the median TyG or TyG-BMI level. TyG, triglyceride-glucose; BMI, Body Mass Index. Model 0: Unadjusted. Model 1: Admission age, gender, race, marital status (The BMI index will be additionally adjusted for the TyG index). Model 2: In addition to the variables in Model 1, add heart rate, SBP, DBP, respiratory rate, temperature. Model 3: In addition to the variables in Model 2, add potassium, creatinine, total bilirubin, LDH, neutrophils, WBC, RBC, platelet, hemoglobin. Model 4: In addition to the variables in Model 3, add APSIII, LODS, SIRS, atrial fibrillation, acute myocardial infarction, congestive heart failure, hypertension, malignant neoplasm, ascites, severe liver disease, COPD, rheumatic disease, hypothyroidism, hyperlipidemia, chronic kidney disease.

Restricted cubic spline analyses were conducted to explore the dose-response relationships between the TyG and TyG-BMI indices and the risk of AP. As shown in Fig. 2A, the TyG index demonstrated a consistent, positive, and approximately linear association with AP risk across all adjusted models (Model 0–Model 4). The overall odds ratios ranged from 1.804 to 1.978 (all *p* < 0.001), indicating that each one-unit increase in the TyG index was associated with an 80.4%–97.8% higher risk of developing AP. Notably, all tests for nonlinearity were nonsignificant (p > 0.05), confirming a stable linear dose-response pattern. These findings highlight the robustness of the TyG index as an independent predictor of AP risk across different adjustment levels. It should be noted that although the different adjustment models are aligned in direction, the fully adjusted model may carry the risk of over-adjustment, the interpretation of association strength should be approached with caution. This study uses Model 2 as the primary inference model, and its results better reflect the early predictive value of TyG as a metabolic risk indicator.

In contrast, the TyG-BMI index exhibited a significant nonlinear relationship with AP risk (Fig. 2B). The nonlinearity p-values for all five models were < 0.001, suggesting a distinct threshold effect. The breakpoints identified by segmented logistic regression ranged from 344.417 to 359.996. Below the BP, the TyG-BMI index was positively associated with AP risk (aOR = 1.008, 95% CI 1.005–1.012), whereas above the BP, the association plateaued or slightly declined (aOR = 0.996, 95% CI 0.991–1.001), indicating that the risk tended to stabilize beyond the threshold.

### Subgroup analyses of the associations between TyG-related indices and the risk of acute pancreatitis

**Fig. 3:**
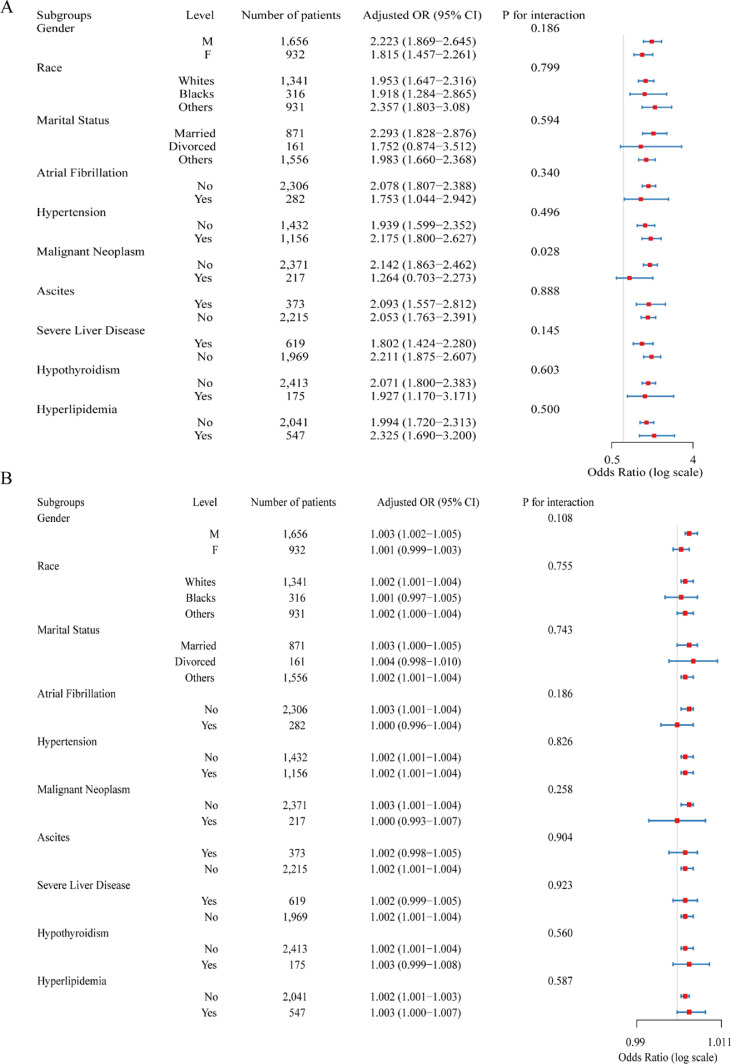
Subgroup analyses of the associations between TyG index, TyG-BMI index, and the risk of acute pancreatitis. (A) Subgroup analysis of the association between the TyG index and the risk of Acute Pancreatitis (AP). (B) Subgroup analysis of the association between the TyG-BMI index and AP risk. The models were adjusted for age, sex, BMI, comorbidities, and laboratory indicators. The horizontal bars represent adjusted Odds Ratios (ORs) with 95% Confidence Intervals (95% CIs). No significant interaction was observed across subgroups, indicating that the associations were consistent among different clinical strata.

Comprehensive subgroup analyses were performed to evaluate the consistency of the associations between TyG and TyG-BMI indices and the risk of AP. As shown in Fig. 3A and Fig. 3B, both indices were positively associated with AP risk across all predefined subgroups, and no significant effect modifications were observed except for malignant neoplasm (p-interactions were > 0.05).

For the TyG index, the positive relationship with AP remained robust and directionally consistent across demographic and clinical strata. The association appeared more pronounced in certain subgroups, particularly among patients with severe liver disease (aOR = 0.802, 95% CI 1.424–2.280), hypertension (aOR = 2.175), and atrial fibrillation (aOR = 1.753). However, no significant heterogeneity was detected by sex, race, or comorbidity (except for malignant neoplasm) (all p for interaction > 0.05).

Similarly, the TyG-BMI index demonstrated a weak but independent positive association with AP risk, which remained consistent across all subgroups. Comparable associations were observed between men and women, across racial and marital categories, and among patients with major comorbidities such as hypertension, atrial fibrillation, and severe liver disease (aOR range: 1.000–1.002).

### Predictive performance of TyG and TyG-BMI indices

**Fig. 4:**
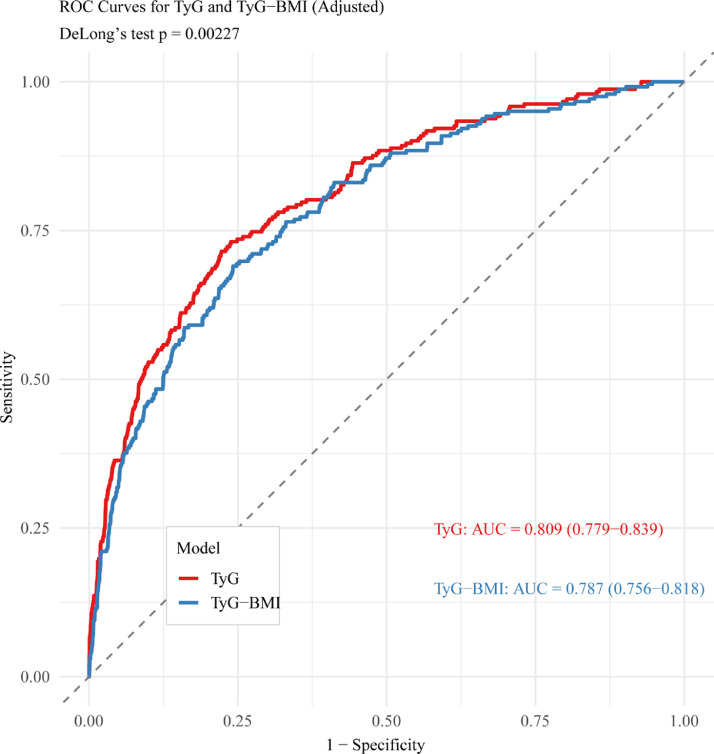
Receiver Operating Characteristic (ROC) curves comparing the predictive performance of the TyG index (red line) and the TyG-BMI composite index (blue line) for acute pancreatitis.

Receiver operating characteristic curve analyses were conducted to compare the predictive accuracy of the TyG and TyG‑BMI indices for acute pancreatitis (Fig. 4). The TyG index demonstrated good discriminative ability, with an AUC of 0.809. In comparison, the TyG‑BMI index showed a slightly lower AUC of 0.787, and this difference was statistically significant according to the DeLong test (*p* = 0.00227). Although the TyG index exhibited statistically higher discriminative performance, the magnitude of the difference between the two indices was modest. These findings indicate that incorporating BMI into the TyG index did not enhance predictive accuracy and may slightly attenuate its performance, suggesting that the TyG index alone provides a marginally better but not substantially superior prediction of AP risk.

### Sensitivity analysis

To assess the robustness of these findings, the authors conducted a series of sensitivity analyses. After excluding patients with hyperlipidemia (n = 547), the association between the TyG index and the risk of acute pancreatitis remained significant (OR = 1.829, 95% CI 1.550–2.157). The authors further excluded individuals with diabetes, and higher TyG levels were similarly associated with an approximately 86.8% increase in the risk of acute pancreatitis. In addition, the authors compared the association between TyG and acute pancreatitis before and after multiple imputation, and the results were highly consistent (OR = 2.09 before imputation vs. OR = 1.87 after imputation), further supporting the stability and robustness of these findings.

## Discussion

In this study of 2,588 adult critically ill patients, the authors demonstrated that elevated TyG and TyG-BMI indices were independently associated with a higher risk of acute pancreatitis. Patients with high TyG or TyG-BMI exhibited more pronounced metabolic disturbances, including hyperglycemia, hypertriglyceridemia, and renal dysfunction, alongside longer ICU and hospital stays. Notably, the TyG index showed a robust, linear dose-response relationship with AP risk, whereas the TyG-BMI index exhibited a nonlinear pattern with threshold effects.

As a biomarker of insulin resistance, the TyG index has been shown in numerous studies to exhibit superior predictive capability compared with the Quantitative Insulin Sensitivity Check Index (QUICKI) and the Homeostatic Model Assessment of Insulin Resistance (HOMA-IR). Owing to its low cost and easy accessibility, the TyG index holds great promise for widespread clinical application.^16^^,^^17^ Previous studies have demonstrated that the TyG index is closely associated with both short- and long-term survival in surgical critical illness, with more pronounced adverse outcomes observed among stroke patients younger than 60-years. Even after comprehensive adjustment for clinical characteristics and laboratory parameters, an elevated TyG index remains an independent predictor of mortality in ICU patients.^18^^,^^19^

Mechanistically, in critically ill patients, an increased TyG index primarily reflects metabolic abnormalities associated with insulin resistance, and it may hypothetically contribute to the development and progression of multiorgan dysfunction.^20^^,^^21^ Critically ill patients frequently exhibit stress-induced hyperglycemia and hyperlipidemia, while the release of catecholamines, cortisol, and inflammatory cytokines such as TNF‑α and IL‑6 further reduces insulin sensitivity and exacerbates metabolic dysregulation.^22^^,^^23^ Thus, an elevated TyG index can be regarded as a comprehensive indicator of stress‑related metabolic imbalance rather than a direct causal factor. Insulin resistance has been linked to chronic low‑grade inflammation and may amplify systemic inflammatory responses through metabolic-immune crosstalk.^24^ Impaired insulin signaling could promote metabolic reprogramming of immune cells, leading to excessive secretion of proinflammatory cytokines such as IL‑1β and IL‑6, thereby forming a hypothetical “metabolic-inflammatory positive feedback loop” that may trigger or magnify cytokine storms in critically ill patients.^25^^,^^26^ In addition, mitochondrial dysfunction and excessive reactive oxygen species production induced by insulin resistance have been proposed to aggravate cellular injury.^27^^,^^28^ While these mechanisms are biologically plausible, they remain speculative in the context of the present study, which is associative in nature and does not provide direct mechanistic evidence.

In our study, even after adjustment for potential confounding factors, each one-unit increase in TyG as a continuous variable was associated with an 89% higher risk of acute pancreatitis among critically ill patients. A similar trend was observed for the TyG-derived index. Previous studies have shown that an elevated TyG-BMI index is associated with an increased risk of in-hospital mortality after severe acute pancreatitis.^29^, ^30^, ^31^ This may be explained by the activation of the renin-angiotensin-aldosterone system and the MAPK signaling pathway under conditions of insulin resistance, which suppresses nitric oxide synthesis and consequently leads to endothelial dysfunction and microcirculatory disturbances.^32^ In critically ill states, such endothelial injury is further amplified, resulting in tissue hypoxia, enhanced oxidative stress, and impaired organ perfusion. Pancreatic microcirculatory dysfunction may therefore exacerbate ischemia–reperfusion injury, promoting the onset and progression of AP.^33^, ^34^, ^35^

The TyG-BMI index has been reported to show higher accuracy than other IR indices and has been widely applied in the assessment of ischemic cerebrovascular events, heart failure, and the need for percutaneous coronary intervention. Yang et al.^36^ reported a nonlinear relationship between the TyG index and in-hospital mortality in acute pancreatitis, with every 10-unit increase in TyG associated with a 5% higher mortality risk. In the present analysis, however, the TyG index demonstrated superior performance over TyG-BMI in predicting acute pancreatitis in critically ill patients (AUC: 0.809 vs. 0.787). Prior studies provide some clues: due to the so-called ‘obesity paradox’, patients with higher BMI or HF tend to have better outcomes than leaner counterparts, suggesting that TyG and BMI may exert opposite prognostic influences. Combining these two measures may therefore obscure true associations, resulting in diminished predictive efficiency for TyG-BMI.^37^^,^^38^ Furthermore, some critically ill patients experience “pseudo-low BMI” owing to malnutrition or hypercatabolism, which can distort BMI-based calculations and weaken the sensitivity of TyG-BMI in reflecting genuine insulin resistance levels.^39^

The consistent associations observed across multiple subgroups, including sex, comorbidity status, and renal function, suggested that TyG was a robust and generalizable metabolic risk marker for AP. Importantly, compared with TyG-BMI, the TyG index exhibited superior predictive performance, implying that a simple biochemical marker of insulin resistance may be more clinically practical than composite anthropometric indices in the intensive care setting. Clinically, these findings underscore the potential utility of early metabolic assessment for identifying high-risk patients. Monitoring TyG levels could facilitate timely interventions aimed at mitigating metabolic stress ‒ such as glycemic control, lipid-lowering therapy, or targeted nutritional support ‒ which may ultimately reduce the incidence or severity of AP. Future prospective studies are warranted to confirm these findings and to elucidate the molecular mechanisms linking insulin resistance and lipid dysregulation to pancreatic injury, including the roles of inflammatory mediators, oxidative stress pathways, and acinar cell apoptosis.

This study has several notable strengths. First, it is based on a large and high‑quality database (MIMIC‑IV), which provides comprehensive clinical, biochemical, and outcome data, allowing for robust adjustment for multiple confounding factors. Second, by focusing on middle‑aged critically ill patients, this study fills an important knowledge gap, as most prior research on the TyG index and metabolic risk has focused on community or cardiovascular populations rather than on critically ill cohorts. Third, the analysis compared the predictive power of both the TyG and TyG‑BMI indices, revealing that the simpler TyG index may outperform its derivative in this special population ‒ an insight that could guide metabolic risk assessment in intensive care practice. Nevertheless, the study is limited by its single‑center, retrospective design, as the MIMIC‑IV database represents one institution (Beth Israel Deaconess Medical Center), which may restrict generalizability and highlights the need for external validation in multicenter cohorts.

In addition, several limitations should be acknowledged. First, the retrospective design and single‑center nature of the MIMIC database limit causal inference and may introduce selection bias. Second, the TyG and TyG‑BMI indices were calculated from single baseline measurements, which may not fully reflect dynamic alterations in glucose and lipid metabolism during critical illness. Third, information on other established markers of insulin resistance, such as HOMA‑IR or the hyperinsulinemic‑euglycemic clamp test, was unavailable, preventing direct comparisons with these reference standards. Fourth, detailed pancreatic imaging and morphological assessments were not accessible, making it impossible to evaluate disease severity based on structural criteria. Fifth, data on alcohol use ‒ an important traditional risk factor and potential confounder for acute pancreatitis ‒ were incomplete in the database and could not be incorporated into the multivariable models, raising the possibility of residual confounding. Finally, because the study population consisted primarily of Western patients from a single institution, external validation in larger and more ethnically diverse cohorts is needed to confirm the generalizability of these findings.

## Conclusion

Both the TyG and TyG‑BMI indices were independently associated with the risk of acute pancreatitis in adult critically ill patients. Between the two markers, the TyG index showed a statistically higher yet modestly improved discriminative performance, accompanied by a clearer linear dose‑response pattern. These findings suggest that TyG may serve as a practical metabolic risk indicator in the intensive care setting, although its incremental advantage over TyG‑BMI is limited in magnitude. Further external validation in multicenter and ethnically diverse cohorts is needed to confirm the generalizability and clinical applicability of these results.

## Ethics approval and consent to participate

This research does not include any research conducted by any author on human participants or animals.

## Consent for publication

Not applicable.

## Data availability statement

Data supporting the findings of this study are available from the Massachusetts Institute of Technology (MIT) and Beth Israel Deaconess Medical Center (BIDMC), but limitations apply to the availability of these data, which were used with permission from the current study and are therefore not public.

## Authors' contributions

M.N.L. and M.L.Z. wrote the manuscript. M.N.L. and Y.P.L. designed the research. L.F.P., Y.A.J. and Z.H. performed the research. M.N.L. and Y.P.L. analyzed the data.

## Funding

Not applicable.

## Declaration of competing interest

The authors declare no conflicts of interest.

## Appendix Supplementary materials

Image, application 1
