# Supplementary material for: Triglyceride-glucose and TyG-BMI indexes predict acute pancreatitis in adult critically ill patients admitted to intensive care
Source: Clinics (Sao Paulo). 2026 Jun 1;81:101007. doi: 10.1016/j.clinsp.2026.101007 (PMC13241886; doi:10.1016/j.clinsp.2026.101007)
Supplement: Supplementary file 1 [file mmc1.docx]

CLINICS-D-25-01748_Supplementary Material

**Table S1** Variables imputed in this study.

| **Variables** | **Column** | **Total** | **na_count** | **non_na_count** | **na_ratio** |
| --- | --- | --- | --- | --- | --- |
| Admission age | Admission age | 2588 | 0 | 2588 | 0 |
| Gender | Gender | 2588 | 0 | 2588 | 0 |
| Race | Race | 2588 | 0 | 2588 | 0 |
| Marital status | Marital status | 2588 | 0 | 2588 | 0 |
| Height | Height | 2588 | 0 | 2588 | 0 |
| Weight | Weight | 2588 | 0 | 2588 | 0 |
| Los hospital | Los hospital | 2588 | 0 | 2588 | 0 |
| Los ICU | Los icu | 2588 | 0 | 2588 | 0 |
| Glucose | Glucose | 2588 | 0 | 2588 | 0 |
| Triglycerides | Triglycerides | 2588 | 0 | 2588 | 0 |
| Heart rate | Heart rate | 2588 | 5 | 2583 | 0.0019 |
| SBP | SBP | 2588 | 5 | 2583 | 0.0019 |
| DBP | DBP | 2588 | 5 | 2583 | 0.0019 |
| Respiratory rate | Respiratory rate | 2588 | 8 | 2580 | 0.0031 |
| Temperature | Temperature | 2588 | 63 | 2525 | 0.0243 |
| Bun | Bun | 2588 | 19 | 2569 | 0.0073 |
| Potassium | Potassium | 2588 | 18 | 2570 | 0.007 |
| Creatinine | Creatinine | 2588 | 18 | 2570 | 0.007 |
| Total bilirubin | Total bilirubin | 2588 | 608 | 1980 | 0.2349 |
| LDH | LDH | 2588 | 1203 | 1385 | 0.4648 |
| Neutrophils | Neutrophils | 2588 | 1234 | 1354 | 0.4768 |
| WBC | WBC | 2588 | 29 | 2559 | 0.0112 |
| RBC | RBC | 2588 | 29 | 2559 | 0.0112 |
| Platelet | Platelet | 2588 | 27 | 2561 | 0.0104 |
| Hemoglobin | Hemoglobin | 2588 | 31 | 2557 | 0.012 |
| APSIII | APSIII | 2588 | 0 | 2588 | 0 |
| LODS | LODS | 2588 | 0 | 2588 | 0 |
| SIRS | SIRS | 2588 | 0 | 2588 | 0 |
| Atrial Fibrillation | Atrial Fibrillation | 2588 | 0 | 2588 | 0 |
| Acute Myocardial Infarction | Acute Myocardial Infarction | 2588 | 0 | 2588 | 0 |
| Congestive Heart Failure | Congestive Heart Failure | 2588 | 0 | 2588 | 0 |
| Hypertension | Hypertension | 2588 | 0 | 2588 | 0 |
| Malignant Neoplasm | Malignant Neoplasm | 2588 | 0 | 2588 | 0 |
| Ascites | Ascites | 2588 | 0 | 2588 | 0 |
| Severe Liver Disease | Severe Liver Disease | 2588 | 0 | 2588 | 0 |
| COPD | COPD | 2588 | 0 | 2588 | 0 |
| Rheumatic Disease | Rheumatic Disease | 2588 | 0 | 2588 | 0 |
| Hypothyroidism | Hypothyroidism | 2588 | 0 | 2588 | 0 |
| Hyperlipidemia | Hyperlipidemia | 2588 | 0 | 2588 | 0 |
| Chronic Kidney Disease | Chronic Kidney Disease | 2588 | 0 | 2588 | 0 |

LDH, Lactate Dehydrogenase; RBC, Red Blood Cell; WBC, White Blood Cell; COPD, Chronic Obstructive Pulmonary Disease; APSIII, Acute Physiology Score III; LODS, Logistic Organ Dysfunction System; SIRS, Systemic Inflammatory Response Syndrome.

**Table S2** Baseline characteristics of middle-aged adults’ populations according to median TyG-BMI index.

|  | **Level** | **Overall** | **High (TyG-BMI index ≥ 271.986)** | **Low (TyG-BMI index < 271.986)** | **p-value** |
| --- | --- | --- | --- | --- | --- |
| **n** |  | 2,588 | 1,294 | 1,294 |  |
| **Admission age (median [IQR], years)** |  | 49.0 [39.2, 54.9] | 49.1 [40.4, 55.2] | 48.8 [37.7, 54.8] | 0.022 |
| **Gender (%)** | Female | 932 (36.0) | 465 (35.9) | 467 (36.1) | 0.967 |
|  | Male | 1656 (64.0) | 829 (64.1) | 827 (63.9) |  |
| **Height (median [IQR], cm)** |  | 173.0 [165.0, 178.0] | 173.0 [165.0, 179.0] | 173.0 [163.4, 178.0] | 0.839 |
| **Weight (median [IQR], kg)** |  | 87.0 [71.8, 104.5] | 104.0 [91.8, 121.8] | 73.0 [63.0, 82.3] | <0.001 |
| **Race (%)** | Blacks | 316 (12.2) | 172 (13.3) | 144 (11.1) | 0.116 |
|  | Others | 931 (36.0) | 474 (36.6) | 457 (35.3) |  |
|  | Whites | 1,341 (51.8) | 648 (50.1) | 693 (53.6) |  |
| **Marital status (%)** | Divorced | 161 (6.2) | 86 (6.6) | 75 (5.8) | 0.316 |
|  | Married | 871 (33.7) | 448 (34.6) | 423 (32.7) |  |
|  | Others | 1,556 (60.1) | 760 (58.7) | 796 (61.5) |  |
| **Los hospital (median [IQR], days)** |  | 13.4 [5.9, 25.0] | 14.2 [6.6, 26.7] | 12.7 [5.5, 23.4] | 0.004 |
| **Los ICU (median [IQR], days)** |  | 6.1 [2.4, 13.3] | 7.0 [2.6, 14.6] | 5.3 [2.2, 12.1] | <0.001 |
| **Heart rate (median [IQR], bpm)** |  | 94.0 [80.0, 110.0] | 96.0 [82.0, 112.0] | 92.0 [78.0, 107.0] | <0.001 |
| **SBP (median [IQR], mmHg)** |  | 124.0 [109.0, 140.0] | 125.0 [109.0, 141.8] | 123.0 [108.0, 140.0] | 0.088 |
| **DBP (median [IQR], mmHg)** |  | 74.0 [63.0, 86.0] | 74.0 [62.0, 86.0] | 74.0 [63.0, 86.0] | 0.239 |
| **Respiratory rate (median [IQR], bpm)** |  | 20.0 [17.0, 25.0] | 21.0 [17.0, 26.0] | 19.0 [16.0, 24.0] | <0.001 |
| **Temperature (median [IQR], °C)** |  | 36.9 [36.6, 37.3] | 36.9 [36.6, 37.3] | 36.8 [36.5, 37.2] | <0.001 |
| **Bun (median [IQR], mg/dL)** |  | 16.0 [11.0, 25.0] | 17.0 [12.0, 28.0] | 14.0 [10.0, 22.0] | <0.001 |
| **Potassium (median [IQR], mEq/L)** |  | 4.1 [3.7, 4.5] | 4.2 [3.8, 4.7] | 4.0 [3.6, 4.4] | <0.001 |
| **Creatinine (median [IQR], mg/dL)** |  | 0.9 [0.7, 1.4] | 1.0 [0.8, 1.7] | 0.9 [0.7, 1.2] | <0.001 |
| **Total bilirubin (median [IQR], mg/dL)** |  | 0.7 [0.4, 1.5] | 0.7 [0.4, 1.5] | 0.7 [0.4, 1.4] | 0.991 |
| **LDH (median [IQR], IU/L)** |  | 354.0 [230.0, 591.0] | 377.0 [245.0, 640.0] | 341.5 [220.0, 545.0] | <0.001 |
| **Neutrophils (median [IQR], %)** |  | 81.0 [73.0, 87.1] | 81.4 [73.9, 87.2] | 81.0 [71.8, 87.0] | 0.088 |
| **WBC (median [IQR], %)** |  | 11.4 [8.0, 16.2] | 12.1 [8.3, 17.2] | 10.8 [7.7, 15.3] | <0.001 |
| **RBC (median [IQR], %)** |  | 4.0 [3.3, 4.5] | 4.0 [3.4, 4.6] | 3.9 [3.2, 4.5] | <0.001 |
| **Platelet (median [IQR], 10^9^/L)** |  | 203.5 [141.0, 267.0] | 207.0 [144.2, 273.0] | 200.0 [136.0, 263.0] | 0.05 |
| **Hemoglobin (median [IQR], g/dL)** |  | 11.8 [9.8, 13.5] | 11.9 [10.0, 13.6] | 11.7 [9.6, 13.4] | 0.039 |
| **APSIII (median [IQR])** |  | 44.0 [30.0, 64.0] | 48.0 [32.0, 70.0] | 40.0 [28.0, 58.0] | <0.001 |
| **LODS (median [IQR])** |  | 5.0 [2.0, 8.0] | 5.0 [3.0, 8.0] | 4.0 [2.0, 7.0] | <0.001 |
| **SIRS (median [IQR])** |  | 3.0 [2.0, 4.0] | 3.0 [2.0, 4.0] | 3.0 [2.0, 3.0] | <0.001 |
| **Glucose (median [IQR], mg/dL)** |  | 129.0 [104.0, 171.0] | 141.0 [112.2, 193.0] | 118.0 [98.0, 150.0] | <0.001 |
| **Triglycerides (median [IQR], mg/dL)** |  | 151.5 [99.0, 263.0] | 213.0 [129.0, 363.8] | 120.0 [83.0, 177.0] | <0.001 |
| **Atrial Fibrillation (%)** | No | 2306 (89.1) | 1120 (86.6) | 1186 (91.7) | <0.001 |
|  | Yes | 282 (10.9) | 174 (13.4) | 108 (8.3) |  |
| **Acute Myocardial Infarction (%)** | No | 2523 (97.5) | 1264 (97.7) | 1259 (97.3) | 0.615 |
|  | Yes | 65 (2.5) | 30 (2.3) | 35 (2.7) |  |
| **Congestive Heart Failure (%)** | No | 2215 (85.6) | 1081 (83.5) | 1134 (87.6) | 0.004 |
|  | Yes | 373 (14.4) | 213 (16.5) | 160 (12.4) |  |
| **Hypertension (%)** | No | 1432 (55.3) | 616 (47.6) | 816 (63.1) | <0.001 |
|  | Yes | 1156 (44.7) | 678 (52.4) | 478 (36.9) |  |
| **Malignant Neoplasm (%)** | No | 2371 (91.6) | 1208 (93.4) | 1163 (89.9) | 0.002 |
|  | Yes | 217 (8.4) | 86 (6.6) | 131 (10.1) |  |
| **Ascites (%)** | No | 2215 (85.6) | 1120 (86.6) | 1095 (84.6) | 0.179 |
|  | Yes | 373 (14.4) | 174 (13.4) | 199 (15.4) |  |
| **Severe Liver Disease (%)** | No | 1969 (76.1) | 992 (76.7) | 977 (75.5) | 0.519 |
|  | Yes | 619 (23.9) | 302 (23.3) | 317 (24.5) |  |
| **COPD (%)** | No | 2462 (95.1) | 1213 (93.7) | 1249 (96.5) | 0.001 |
|  | Yes | 126 (4.9) | 81 (6.3) | 45 (3.5) |  |
| **Rheumatic Disease (%)** | No | 2524 (97.5) | 1269 (98.1) | 1255 (97.0) | 0.1 |
|  | Yes | 64 (2.5) | 25 (1.9) | 39 (3.0) |  |
| **Hypothyroidism (%)** | No | 2413 (93.2) | 1201 (92.8) | 1212 (93.7) | 0.434 |
|  | Yes | 175 (6.8) | 93 (7.2) | 82 (6.3) |  |
| **Hyperlipidemia (%)** | No | 2041 (78.9) | 981 (75.8) | 1060 (81.9) | <0.001 |
|  | Yes | 547 (21.1) | 313 (24.2) | 234 (18.1) |  |
| **Chronic Kidney Disease (%)** | No | 2345 (90.6) | 1159 (89.6) | 1186 (91.7) | 0.080 |
|  | Yes | 243 (9.4) | 135 (10.4) | 108 (8.3) |  |
| **Acute Pancreatitis (%)** | No | 2,346 (90.6) | 1,138 (87.9) | 1,208 (93.4) | <0.001 |
|  | Yes | 242 (9.4) | 156 (12.1) | 86 (6.6) |  |
| **TyG (median [IQR])** |  | 9.3 [8.7, 9.9] | 9.7 [9.1, 10.3] | 8.9 [8.5, 9.4] | <0.001 |
| **BMI (median [IQR], kg/m^2^)** |  | 29.3 [25.0, 34.7] | 34.7 [31.5, 41.0] | 25.0 [22.3, 27.4] | <0.001 |

TyG, Triglyceride-Glucose; IQR, Interquartile Range; BMI, Body Mass Index; LDH, Lactate Dehydrogenase; RBC, Red Blood Cell; WBC, White Blood Cell; COPD, Chronic Obstructive Pulmonary Disease; APSIII, Acute Physiology Score III; LODS, Logistic Organ Dysfunction System; SIRS, Systemic Inflammatory Response Syndrome.
